# Wunderlich Syndrome in a Patient With Type 1 Diabetes Mellitus: Surgical Challenges and Management Strategies

**DOI:** 10.7759/cureus.50481

**Published:** 2023-12-13

**Authors:** José Luis Serafio-Gómez, Carlos Ulises Anderson-Flores, Alejandra María Valenzuela-Leal, Daniel Tarin-Recendez, Roberto Pastor-Andujo, José F. De la Torre-Ramos

**Affiliations:** 1 General Surgery, Chihuahua City General Hospital “Dr. Salvador Zubirán Anchondo”, Chihuahua, MEX; 2 General Surgery, Chihuahua City General Hospital "Dr. Salvador Zubirán Anchondo", Chihuahua, MEX

**Keywords:** multidisciplinary management, laparoscopic drainage, surgical intervention, conservative management, angiomyolipoma, spontaneous renal hemorrhage, wunderlich syndrome

## Abstract

Wunderlich syndrome, a rare manifestation of spontaneous renal hemorrhage often attributed to renal angiomyolipomas, presents a complex clinical scenario demanding nuanced management. This article delves into the multifaceted dimensions of this syndrome, dissecting its clinical, diagnostic, and therapeutic intricacies. Illustrating this complexity is the case of a 26-year-old female with Wunderlich syndrome and comorbid type 1 diabetes mellitus, revealing challenges at the intersection of these conditions.

While initial intervention via laparoscopic drainage and antibiotic therapy yielded symptomatic relief, the subsequent recurrence of a renal abscess prompted a re-evaluation of the treatment strategy, culminating in a second surgical intervention. The intricate interplay between Wunderlich syndrome and diabetes introduces unique challenges, with fluctuations in hemoglobin and recurrent leukocytosis mirroring the underlying complexities of this clinical dyad.

This case underscores the indispensability of a multidisciplinary approach, seamlessly integrating medical and surgical modalities, coupled with vigilant postoperative monitoring. Swift identification of complications and adaptability of the treatment plan emerged as pivotal in addressing recurrent manifestations and averting long-term sequelae. The necessity for continuous surveillance and personalized management strategies becomes evident, emphasizing Wunderlich syndrome as a clinical entity requiring bespoke attention.

In conclusion, this case serves as an example, highlighting the intricate nature of Wunderlich syndrome, accentuated by the presence of type 1 diabetes mellitus. The initial therapeutic success, followed by a recurrence, underscores the need for ongoing research, paving the way for refined diagnostic and treatment paradigms. The synthesis of clinical complexities in this scenario elucidates the imperative for a comprehensive understanding, guiding future endeavors aimed at optimizing the prognosis of patients affected by this uncommon syndrome.

## Introduction

Wunderlich syndrome, also known as spontaneous renal hemorrhage, is a rare yet potentially serious clinical entity characterized by non-traumatic bleeding in the perirenal space. First described by Carl Reinhold August Wunderlich in 1856, it is most commonly associated with the rupture of renal angiomyolipomas [1-7]. However, it can also be linked to other vascular or neoplastic lesions.

Affected patients typically present with abrupt symptoms such as acute abdominal pain, hypovolemic shock, and, in some cases, a palpable mass in the flank. Swift identification and management of this condition are essential to prevent severe complications and preserve renal function [8,9]. Diagnosis is typically achieved through imaging techniques such as abdominal CT, which reveal the presence of blood in the perirenal space. Therapeutic approaches can range from conservative strategies, like observation and supportive therapy, to more invasive interventions, including selective arterial embolization or surgery. The choice of treatment depends on factors such as the underlying cause, hemodynamic stability, and the extent of bleeding.

Although Wunderlich syndrome is uncommon, early recognition and appropriate treatment are crucial for improving clinical outcomes [10,11]. This report explores the clinical, diagnostic, and therapeutic aspects associated with this condition, providing a more comprehensive understanding of its management in contemporary medical practice.

## Case presentation

**Figure 1:**
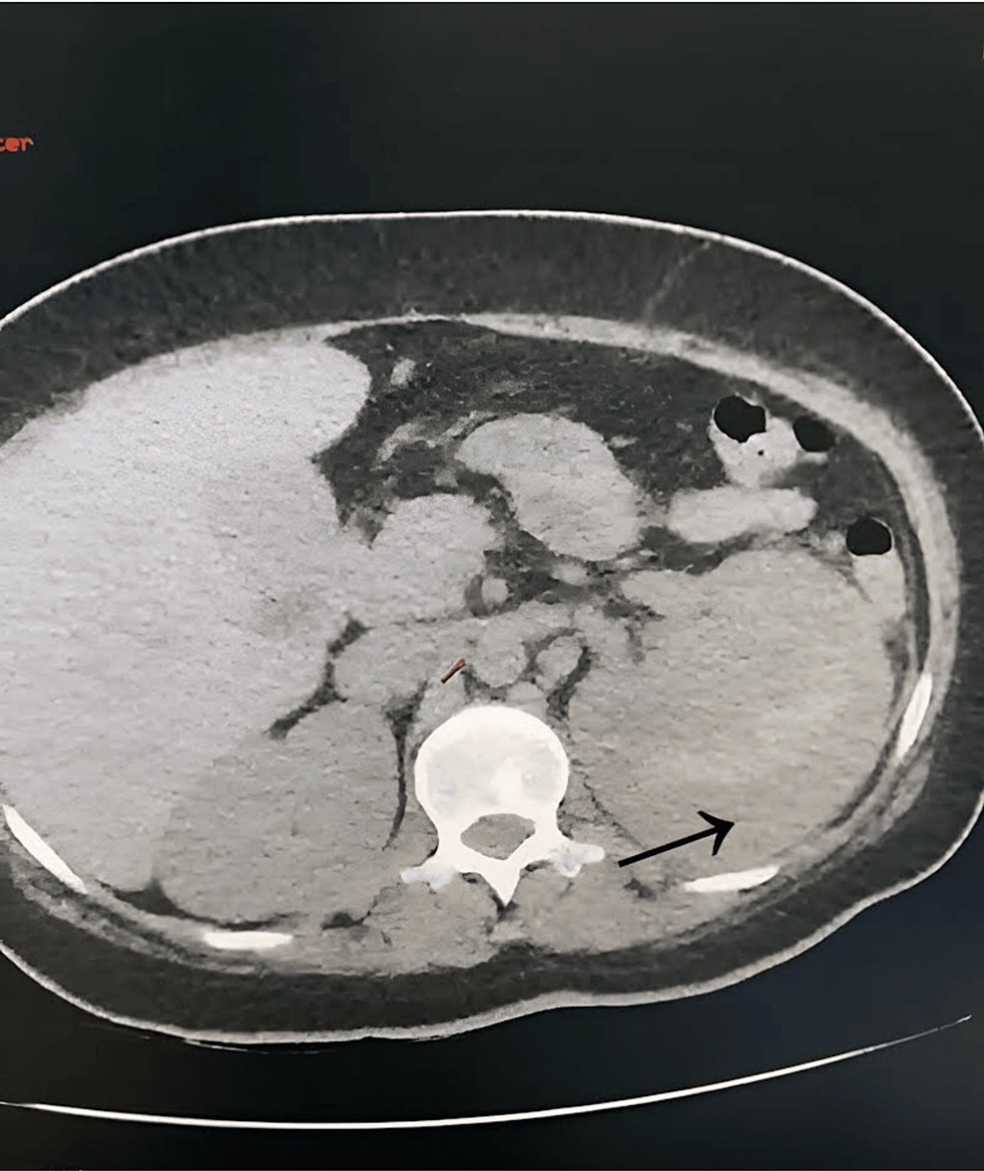
CT scan reveals the presence of a perirenal hematoma (black arrow) surrounding the kidney

The patient was a 26-year-old female with type 1 diabetes mellitus diagnosed at the age of 14, who was under treatment with 10 units of insulin glargine every 24 hours. Upon admission to the emergency room of this hospital, she presented with persistent, lancinating pain for a duration of five days in the left renal fossa that was rated at 7/10 on the visual analog scale (VAS), described as pulsatile and colicky, radiating to the left flank and left inguinal fossa. The pain improved with the intake of non-steroidal anti-inflammatory drugs (NSAIDs). She also reported the presence of unquantified febrile spikes, macroscopic hematuria, nausea, and vomiting on multiple occasions, with gastrointestinal characteristics seemingly unrelated to trauma. On arrival, she had a body temperature of 38 °C, a pulse of 110 heart rate per minute, a respiration rate of 28 respiratory rate per minute, and a blood pressure of 100/60 mmHg. A CT scan revealed a left perirenal lesion involving the left renal parenchyma, without evidence of urinary obstruction or hydronephrosis (Figure 1). It was determined to be the presence of a left renal abscess vs spontaneous hematoma. Laboratory studies conducted upon admission are included in Table 1.

**Table 1:** Laboratory values of the patient upon admission

Laboratory	Reference range*	Patient value	High: H, Low: L
Glucose	74-106 mg/dL	141 mg/dL	H
Urea	12.6-54 mg/dL	30 mg/dL	-
Uric acid	3.5 – 8.5 mg/dL	4.5 mg/dL	-
Creatinine	0.5 -1 mg/dL	0.5 mg/dL	-
Urea nitrogen	7 – 17 mg/dL	12 mg/dL	-
Chloride	98-111 mmol/L	103 mmol/L	-
PCR	0 – 0.1 mg/dL	19.6 mg/dL	H
Potassium	3.5-5.1 mmol/L	5 mmol/L	-
Sodium	137-145 mmol/L	137 mmol/L	-
Calcium	8-10mg/dL	9 mg/dL	-
Magnesium	1.8-3 mg/dL	1.9 mg/dL	-
Hemoglobin	13- 17 g/dL	10.3g/dL	L
Hematocrit	42-50%	32.5%	L
Leukocytes	4.50-11 K/uL	12.6	H
Platelets	150-450k uL	773k/uL	H
Prothrombin time	11-14 sec	14.3 sec	H
INR	1	1.21	H
Activated partial thromboplastin time	20-45 sec	28.1 sec	-

Surgical intervention was deemed imperative upon the revelation of a notable decline in hemoglobin to 8 within 24 hours, coupled with an observed escalation in heart rate to 120 beats per minute. Urgent laparoscopic surgery was initiated, notwithstanding the initiation of packed red blood cell transfusion, yielding only a marginal 0.5 increment in the hemoglobin range.

**Figure 2:**
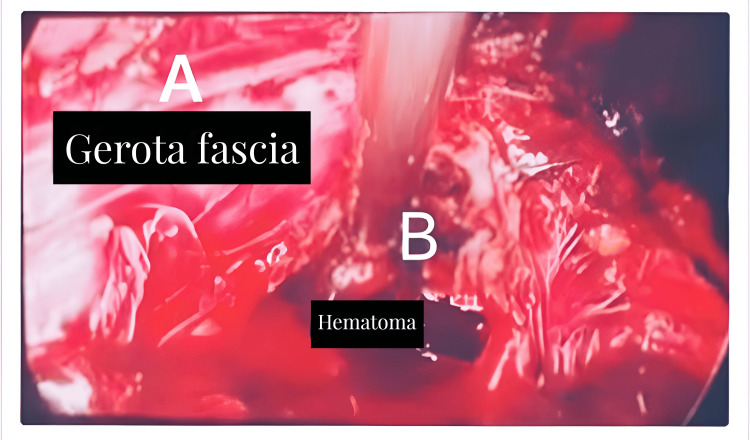
Laparoscopic drainage of abscessed hematoma

The laparoscopic drainage procedure addressed the abscessed hematoma in the left kidney. During this intervention, retroperitoneal access was achieved, delineating the lower pole of the left kidney. Subsequent to identifying Gerota's fascia, it was incised using laparoscopic scissors, resulting in the immediate drainage of approximately 400 cc of purulent material. A subsequent incision, measuring approximately 3 cm, facilitated access to the perirenal cavity. Within this cavity, the majority of clots were extracted, accompanied by surgical cleansing and the placement of a Saratoga-type drainage. This approach was dictated by the available resources in the hospital, where essential materials for percutaneous drainage and embolization were regrettably unavailable (Figure 2).

Post-surgery, antibiotic therapy was sustained, two units of red blood cell concentrates were transfused, and clinical monitoring persisted over the subsequent three days. The absence of leukocytosis and elevation of hemoglobin levels to within normal ranges were noted, prompting the decision to discharge the patient.

**Figure 3:**
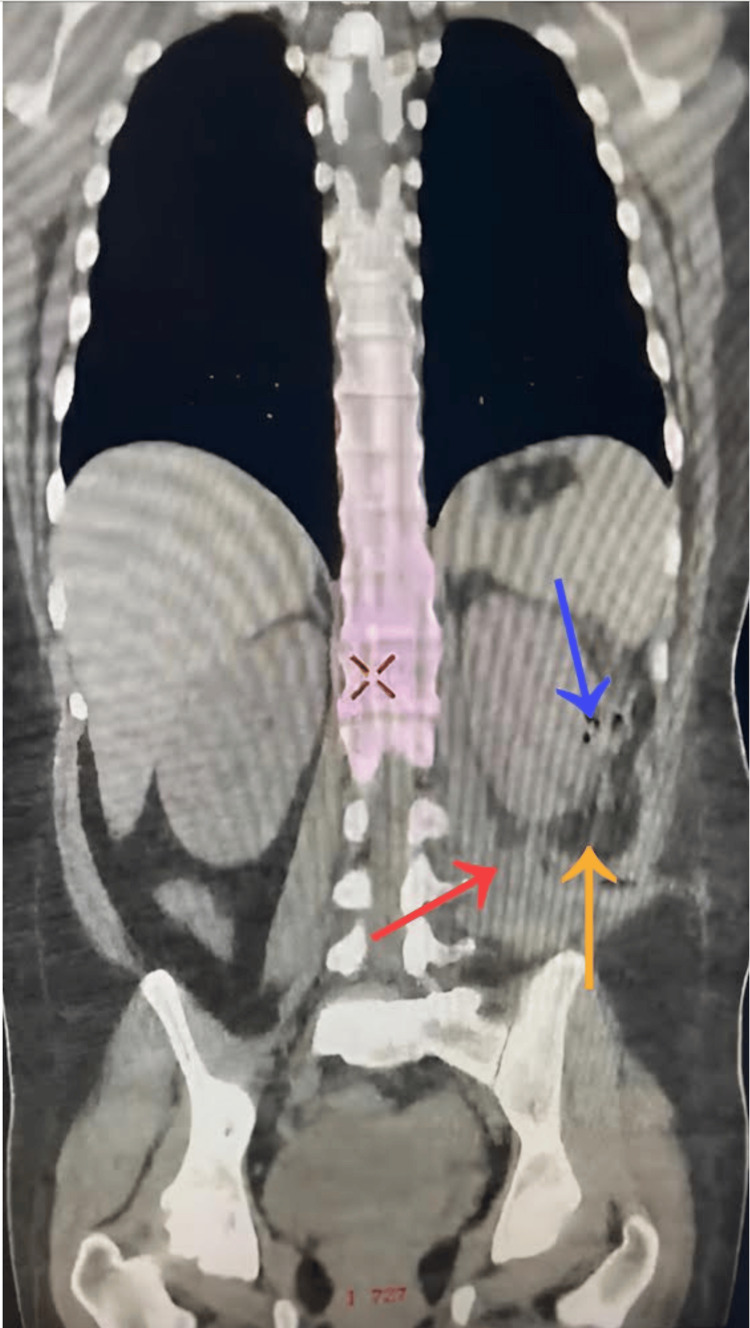
Post-surgical CT revealing residual abscess Fluid accumulation (red arrow) is seen in the left renal fossa, accompanied by emphysema (blue arrow) and a slight amount of striated adipose tissue (yellow arrow).

Four weeks following her hospital discharge, the patient presented with leukocytosis of 12.4, febrile episodes, and the discharge of purulent and malodorous material through a Saratoga-type drainage. This scenario, indicative of a postoperative perirenal abscess, constitutes one of the anticipated complications linked to any infected surgical procedure. In this particular instance, it was a consequence of the laparoscopic drainage of the abscessed hematoma. Subsequently, readmission was deemed necessary. A CT scan identified a persistent abscess in the left kidney, necessitating a return to the operating room for abscess drainage via lumbotomy. Simultaneously, a microbiological culture with antibiotic susceptibility testing identified *Escherichia coli,* prompting the initiation of a directed antibiotic regimen with meropenem (Figure 3). Currently, the patient is in a satisfactory overall condition, as per the follow-up consultation held with her three weeks after her last hospitalization, with no recurrence of abscess or spontaneous bleeding.

## Discussion

In this investigation, we observed a noteworthy antecedent of infection, identified as one of the uncommon etiologies contributing to Wunderlich syndrome. The surgical intervention chosen was guided by the available resources at the hospital. This case, where the patient is afflicted by Wunderlich syndrome and type 1 diabetes mellitus, prompts contemplation of various clinical and therapeutic considerations. The confluence of both conditions underscores the intricacies inherent in managing this infrequent pathology [1-4].

The decision to execute laparoscopic drainage of the abscessed hematoma, followed by antibiotic therapy and vigilant monitoring, initially yielded symptomatic amelioration. However, the recurrence of the renal abscess underscores the imperative for sustained follow-up and adaptability in refining the treatment regimen.

In our case, the presence of diabetes mellitus serves as a notable antecedent, coupled with the identification of an abscessed hematoma during surgical intervention. It is of note that such a hematoma constitutes an infrequent etiological factor contributing to the manifestation of Wunderlich syndrome, a clinical condition characterized by non-traumatic renal bleeding into the perirenal space. This association underscores the significance of meticulous clinical evaluation and consideration of less common contributors to renal complications in patients with pre-existing conditions such as diabetes mellitus.

A multidisciplinary approach, encompassing both medical and surgical domains, is indispensable. Swift recognition of complications and nimble adjustments to the therapeutic strategy played a pivotal role in optimizing clinical outcomes [5-12]. This case underscores the urgency for further investigation and comprehension of the interplay between Wunderlich syndrome and type 1 diabetes mellitus. The formulation of precise guidelines for managing analogous cases holds the potential to refine clinical decision-making and, ultimately, enhance the prognosis of afflicted patients [13].

## Conclusions

In this particular case, the determinative etiology was found to be infectious. It is noteworthy to emphasize that the identified hematoma abscess was devoid of any antecedent trauma or discernible etiological factors, underscoring its infectious nature. It is crucial to acknowledge the inherent limitations of this study, primarily stemming from the notably constrained resources available within the hospital.

This case illuminates the intricate and challenging facets associated with Wunderlich syndrome, a condition delineated by spontaneous renal hemorrhage. The complicating factor of type 1 diabetes mellitus as a comorbidity adds a layer of complexity to clinical management. This underlying factor emerges as an atypical etiological contributor to Wunderlich syndrome, a phenomenon characterized by non-traumatic renal hemorrhage into the perirenal space.

The initial therapeutic approach, encompassing medical treatment and laparoscopic drainage, yielded favorable results by transiently alleviating acute symptoms. The subsequent trajectory of the case sheds light on the persistent nature of the issue, manifested by the recurrence of the renal abscess, necessitating a secondary surgical intervention. 

The need for continuous monitoring and a multidisciplinary approach, encompassing medical, surgical, and postoperative follow-up components, is manifest in this case. Swift identification of complications and the adaptive modulation of the treatment plan were pivotal in addressing recurrences and mitigating potential long-term complications.

This case underscores the paramount importance of considering Wunderlich syndrome as a clinical entity necessitating a bespoke approach and continual assessment. Furthermore, it accentuates the indispensability of scrupulous clinical attention in conjunction with the critical importance of sustained research and comprehensive studies to deepen the comprehension of this rare pathological condition. The overarching objective of these research endeavors must be to refine both diagnostic modalities and therapeutic strategies, with the ultimate aim of attaining a treatment regimen that is not only more effective but also conducive to heightened success.

The consideration of therapeutic modalities, such as percutaneous drainage and embolization, becomes highly pertinent. Especially as these interventions possess the inherent potential to not only ameliorate the duration of hospitalization but also contribute significantly to the reduction of post-surgical complications. This strategic approach gains fortification through the application of a conservative management strategy underpinned by antibiotic therapy and vigilant clinical surveillance. 
