# Correction: Wunderlich Syndrome in a Patient With Type 1 Diabetes Mellitus: Surgical Challenges and Management Strategies

**DOI:** 10.7759/cureus.c177

**Published:** 2024-05-24

**Authors:** José Luis Serafio-Gómez, Carlos Ulises Anderson-Flores, Alejandra María Valenzuela-Leal, Daniel Tarin-Recendez, Roberto Pastor-Andujo, José F. De la Torre-Ramos

**Affiliations:** 1 General Surgery, Chihuahua City General Hospital “Dr. Salvador Zubirán Anchondo”, Chihuahua, MEX; 2 General Surgery, Chihuahua City General Hospital "Dr. Salvador Zubirán Anchondo", Chihuahua, MEX

The authors list of this article has been corrected to include senior author José F. de la Torre-Ramos, who was mistakenly omitted from the original author list. Dr. de la Torre-Ramos has been added as the last and corresponding author. The authors greatly regret that this omission was not identified and corrected prior to publication.

